# Health Equity in a Post ‘Roe Versus Wade’ America

**DOI:** 10.7759/cureus.32100

**Published:** 2022-12-01

**Authors:** Jessica J Byron, Melba Avalos, Kexin (Amy) Xiao, Arthur A Klein, Joerg R Leheste

**Affiliations:** 1 Biomedical Sciences, New York Institute of Technology College of Osteopathic Medicine, Jonesboro, USA; 2 Cardiology/Pediatrics, New York Institute of Technology College of Osteopathic Medicine, Old Westbury, USA; 3 Biomedical Sciences, New York Institute of Technology College of Osteopathic Medicine, Old Westbury, USA

**Keywords:** abortion tourism, health equity, social determinants of health (sdoh), supreme court, roe v. wade, sexual and reproductive health rights policy, public health, elective abortion, abortion, abortion laws and society

## Abstract

For almost five decades, the US Supreme Court had protected the right to abortion care in the United States. However, the Court’s decision in ‘Dobbs versus Jackson Women’s Health Organization’ in 2022 established that the US Constitution does not confer a right to abortion, effectively overturning the Court’s own previous judgment in ‘Roe versus Wade’ from 1973. How could a decision that overturns the precedent for nationwide access to abortion affect women, children, and physicians? How will state laws impact healthcare equity regarding reproductive rights going forward? How will geography affect who can access care financially, and how will this shape the conditions that unwanted children are born into? This work is a systematic analysis of the impact of overturning ‘Roe versus Wade’ considering the scientific and medical evidence as well as the conflicting political and moral viewpoints regarding abortion. Furthermore, we make constructive recommendations with a view to detoxifying the inflammatory rhetoric surrounding this topic and protecting key stakeholders such as patients seeking abortion and their physicians.

The moment ‘Roe v. Wade’ was overturned, several US states began to enact laws, both protecting and restricting abortion. California and New York, for example, have since enacted laws that extend protection to both patients seeking abortion and the physicians who provide it. Oklahoma and Texas, on the other hand, have enacted laws that make it more difficult for patients to have abortion access, even after fetal death or when the birthing individual’s life is in danger. These differences are likely to exacerbate the healthcare access divide across the country and increase the financial burden for those who can become pregnant. As differences in healthcare access and quality of care increase across the nation, an unmatched demand for maternity care, especially in the most restrictive states, could precipitate increased maternal and fetal morbidity and mortality while physician shortages are expected to worsen. It will be up to the US Congress to address these differences while weighing competing stakeholder interests. Currently, the nation is at the center of a seismic shift surrounding health policy with laws changing rapidly. This systematic health policy analysis paper will explore what the long-term consequences of this recent Supreme Court decision might look like while considering some of the most recently reported short-term consequences after the landslide decision.

## Introduction and background

On June 24, 2022, the US Supreme Court overturned 49 years of precedent and ended nationwide abortion rights in its decision in ‘Dobbs versus Jackson Women’s Health Organization’ by striking down ‘Roe v. Wade’ and returning regulatory authority back to the individual states [1]. While this decision does not translate into a nationwide ban on abortions, it creates a patchwork of state abortion laws and significant health inequities for birthing individuals across the country. So far, 27 states have chosen to ban or significantly restrict abortion, including Oklahoma, where the governor has signed a ban making it illegal from the moment of conception [2], and Ohio, which enacted laws making abortion illegal at six weeks of gestation, including for victims of rape and incest [3].

In the wake of this landmark Supreme Court decision, the realm of abortion laws in the United States is rapidly evolving and the consequences can be already felt. A Tennessee woman, for example, had to travel six hours by ambulance to receive an abortion after her blood pressure had risen to a level that put her life in jeopardy [4]. Walgreens and CVS have changed their store policies now allowing pharmacists to deny women medications that might trigger an abortion. Whether this constitutes discrimination is currently under investigation by the federal government [5]. Georgia has extended personhood rights to fetuses at six weeks of pregnancy, raising concerns about criminalizing the behavior of pregnant people [6]. Several states will soon vote on upcoming ballot measures that would protect abortion rights or allow legislatures to further restrict them based on the will of the people [7]. Just a few weeks from the conclusion of our analysis, these issues may have already been resolved while others will have arisen.

Unequal access to abortion care has been associated with adverse outcomes for both birthing individuals as well as their children as previously documented by the ‘Turnaway Study’ - the most recent investigation into abortion inequality in the US [8]. With this systematic analysis, we focus attention on the potential outcomes of unequal state abortion regulations based on previous experiences and current trends. America has not faced such challenges in half a century and decisions made in the next few months will be pivotal to women’s and children’s health going forward.

## Review

Historical and current policies surrounding abortion

On March 19, 2018, and as a precursor of more recent events, the state of Mississippi enacted the ‘Gestational Age Act’ into law, banning abortions after 15 weeks of pregnancy, excluding medical emergencies or fetal abnormalities [9]. On the same day, Jackson Women’s Health Organization, Mississippi’s only licensed abortion clinic, challenged the constitutionality of this new law, which was followed by a favorable ruling by the US District Court for the Southern District of Mississippi confirming unconstitutionality. The decision was based on the precedent set by the US Supreme Court’s ‘Roe v. Wade’ decision in 1973 and ‘Planned Parenthood versus Casey’ two decades later. Both rulings stated that states do not have the authority to ban abortions before the 24th week of pregnancy - the time when a fetus is deemed viable [10]. In the wake of these landmark decisions, in 1973 and 1992 respectively, women had the power to make autonomous decisions about their sexual and reproductive health for the first time in American history [11].

Thomas Dobbs, a Mississippi Department of Health officer, pleaded the case further to the US Court of Appeals for the 5th Circuit - a federal court with appellate authority [12]. The ruling confirmed the previous decision by the US District Court for the Southern District of Mississippi in favor of Jackson Women’s Health. On June 15, 2020, Dobbs escalated his appeal to the US Supreme Court, which was granted a review on May 17, 2021, with a hearing scheduled for December 1, 2021. As a result, the Supreme Court ruling on June 24, 2022, finally overturned ‘Roe versus Wade’ by holding that there was no longer a federal constitutional right to abortion, thereby shifting authority to the individual states [13,14]. In anticipation of the Supreme Court ruling, many states had preemptively passed much stricter, so-called ‘trigger laws’ on abortion, which would take immediate effect once the Supreme Court overturned ‘Roe v. Wade’ [15].

In May 2022, an unprecedented leak of Supreme Court judge Sam Alito's draft opinion indicated that most judges voted in favor of overturning the ‘Roe versus Wade’ ruling from 1973, thereby challenging its constitutionality and returning the responsibility back to the individual states [16]. The leaked draft further corroborates this stance by stating that this would empower voters and allow them to elect representatives who align with their views on abortion laws and rights, including women, who would be allowed to vote on policies that impact their reproductive health [16].

With its recent decision, the Supreme Court argues that abortion is not specifically mentioned in the US Constitution, and thus history must be examined to decide whether the Founding Fathers would have considered a right to abortion. This static view calls into question any societal advancements since the adoption of the Constitution. At the time the Constitution was adopted, abortion was illegal in all states with varying degrees of punishment. Thus, the historical analysis clearly suggests that abortion is not protected by the Constitution. Congress must now weigh this claim by the Supreme Court against current standards for human rights [17]. The United Nations asserts that abortion is a human right and there are already humanitarian charities established in Europe to help American women access abortion care anticipating a scenario where it becomes illegal in many US states. Many developed nations view the recent shift away from abortion access in the US as a humanitarian crisis to be concomitant with a general shift in perspective for many American citizens [18]. The United States has long been seen as a leader in human rights. The recent Supreme Court decision may be interpreted as a withdrawal from this position and lower this nation’s political standing in the eyes of the rest of the world.

In response to an increasingly restrictive environment, abortion right supporters have proposed the passing of the Women's Health Protection Act (H.R.3755) under the 117th Congress (2021-22), seeking to codify nationwide abortion protections and outlaw governmental measures that would enforce abortion restriction or limit physicians’ ability to provide abortion care [19]. The bill has been passed by the House of Representatives and is currently held in the Senate. Furthermore, eight states have already expanded access to abortion healthcare and several others are in line for similar bills, including California, Oregon, and Washington [20]. On March 22, 2022, the California Senate passed the Abortion Accessibility Act (S.B.245), prohibiting health plans, including Medicaid, from imposing out-of-pocket costs for abortion services [14]. Additionally, the Committee on Women and Gender Equity of the New York City Council proposed a legislation package seeking to improve abortion access, including the enforcement of prohibitions against city interference, and the provision of funding for New Yorkers and those traveling from out-of-state to obtain safe abortion care [21].

On July 11, 2022, President Biden signed an executive order directing the Secretary of Health and Human Services (HHS) to undertake action to expand access to abortion care, promote education on reproductive healthcare services, and protect pregnant people’s safety and security through access to abortion medication pills, and contraceptive services [22]. Subsequently, on August 3, 2022, President Biden signed a second executive order encouraging states that have not outlawed abortion to apply for Medicaid waivers to help women traveling between states seeking abortion procedures. Many abortion advocates have pointed out that these executive orders will not be sufficient to offset the overruling of ‘Roe v. Wade’. They have urged President Biden to provide concrete plans and actions to address the core issue at stake: women’s equality in reproductive health [23].

As intended by the Supreme Court, Kansas was the first state to test the desire of voters toward more restrictive abortion healthcare. On August 2, 2022, Kansans voted with a majority of 59% to 41% not to allow the state legislature to introduce restrictions to abortion healthcare. For women in Kansas, this means that abortion care is protected until 22 weeks of gestation, ensuring that there is sufficient time to make the most appropriate choice [24]. Kansas’ vote is consistent with historical trends surrounding ballot initiatives on abortion. In 2011, Mississippi voted on a personhood amendment, which would have declared that life begins at fertilization. That amendment was defeated by a 58% to 42% vote [25].

Health impact

According to a survey assessing physicians’ views on abortion policies, legislation banning or substantially limiting abortion could broadly impact a state’s healthcare system [26]. Most healthcare providers across specialties oppose restrictions on abortion care services and policies that would limit a physician’s ability to perform abortion care. Furthermore, the shortage of reproductive healthcare providers raises concerns about the negative impact of the current ruling on the ability of medical institutions in restrictive states to hire and retain physicians [11].

Another expected outcome is an increase in live births triggering additional demands on maternal and obstetric services in areas where abortion is heavily regulated or completely banned. This projection comes amid half of all rural hospitals currently closing down their obstetrics units, further limiting care for pregnant individuals [27]. A 2020 report from the March of Dimes found that seven million American women are living in areas with limited or no access to quality maternal care [28]. At the same time, a major contributor to low birth weight and increased risk of neonatal health complications is the lack of quality healthcare access [29]. Brock Slabach, the chief operations officer at the National Rural Health Association, concluded that the recent Supreme Court decision may be destined to overwhelm the hospitals in rural communities that still deliver babies and offer obstetric services [27]. Many of these hospitals are already facing shortages in healthcare providers, staff, and other delivery resources. A significant influx of new patients would stretch these resources even thinner and eventually reduce the overall quality of care for all patients. Pregnant women living in rural areas will face unprecedented barriers to maternal care and be less likely to seek them as a consequence. Women in labor are projected to travel long distances to hospitals that offer labor and delivery services [30]. In the case of a miscarriage or septic abortion, immediate medical care is crucial to prevent infection, hemorrhage, and maternal death [31]. Barriers to access will disproportionally harm less affluent populations living in areas with little to no access to quality care, resulting in disproportionate risks of maternal complication and fatality [32]. The reversal of ‘Roe v. Wade’ will significantly curtail women’s reproductive rights, access to quality reproductive care, and quality of health. Ultimately, it will exacerbate current shortcomings in healthcare and healthcare delivery, steering affected individuals and their families into a new health crisis [30].

States with the most severe abortion restrictions tend to have the poorest maternal and neonatal health outcomes [33]. Currently, Medicaid covers 41% of all pregnancies, reflecting the reliance of less affluent women on federal and state Medicaid programs for safe and healthy delivery. In this context, it will be particularly important to understand how birthing individuals across the nation are affected by the social determinants of health (SDOH), such as wealth, race and ethnicity, education, and location, after the Supreme Court ruling. For example, women in states where abortion is outlawed may not be able to afford to travel to a different state for abortion care because of the associated transportation costs. This, in turn, would disproportionately disadvantage women in less affluent areas who are not only facing the immediate consequences of unwanted pregnancy but also the long-term economic hardships of raising a child in poverty. Additional children born from unwanted pregnancies will likely add more strain to an already overwhelmed foster care system, further compromising child welfare. These are all potential consequences that must be considered.

It is important to note that pregnancy itself carries the risk of temporary or lifelong adverse health consequences including gestational diabetes, preeclampsia, rhesus factor (Rh) incompatibility reactions, and the worsening of chronic diseases [34]. It also includes an increased risk of death [34]. The United States has a maternal mortality rate higher than much of the rest of the developed world, at 23.8 maternal deaths per 100,000 births. That is more than twice as many as France and Canada and more than three times that of the United Kingdom [35]. In addition to health consequences, pregnancy can also be associated with socioeconomic consequences for career attainment or advancement. Despite the presence of laws to prevent discrimination, it is not unusual for women to experience career setbacks due to having children and even the potential thereof [36,37]. Although the stigma against single motherhood has diminished in the last 50 years, this is not the case for teenage motherhood, and a high school student who finds herself pregnant may find herself judged and pressured to make decisions such as giving her child up for adoption or terminating her pregnancy [38].

Restricting access to abortion care at the state level may increase the risk of total maternal mortality (TMM). States with more restrictive abortion policies show an average TMM increase of 7% compared to those states with less restrictive abortion policies [39]. The US is already among a small cohort of nations that have introduced new restrictions to abortion in the past 28 years; other countries include Poland, El Salvador, and Nicaragua. In contrast, more than 40 nations have expanded abortion rights during that same period [40]. A study from Poland, published in 2021, found that legally imposed restrictions do not change the number of abortions, but they do lead to an increase in mortality resulting from illegal attempts at pregnancy terminations. Affected women are also much more likely to engage in abortion tourism, by seeking help in neighboring countries with less restrictive abortion laws [41].

Abortion can also relieve the crisis of infant mortality. Between 1970 and 1972, the most important factor associated with declining neonatal mortality (death in the first 28 days following birth) among both black and white infants was the increasing availability of induced abortion care [42]. In 2020, the infant mortality rate in the US was 5.7 deaths per 1,000 live births versus 20.5% in 1970 [43]. In 1972, the year before ‘Roe v. Wade’ sanctioned abortion at the federal level, the maternal mortality rate was 18.71 per 1,000 live births versus 8.6 in 2020 [44], indicating that abortion access is a key driver of neonatal and maternal mortality.

Fertility treatment as part of reproductive healthcare is also likely to be impacted. The future of in vitro fertilization (IVF) as a family planning tool for the infertile community has come into question as the definition of life will be up to the state’s discretion. For example, Oklahoma considers fertilization to be the start of a new life and has banned abortion at this point, therefore frozen fertilized embryos would be protected [45]. Such an action could put fertility treatment further out of reach for less affluent patients because embryos would need to be created and stored indefinitely, or, if a patient was unable to comply, undergo the intensive egg retrieval process for each cycle of IVF attempted. This would significantly increase the cost of the procedure and lead to subsequent complications in cases where frozen embryos, for reasons other than immediate infertility treatment, would be needed later. This includes individuals with cancer who fear the adverse effects of chemotherapy and/or radiation treatment on their fertility. If IVF is restricted or outlawed, this could negatively impact working women who choose to delay family planning in lieu of building a career first without dependence on government assistance to financially care for the child. In 2018, an analysis revealed that Medicaid paid for a greater share of births among women under the age of 19 [46]. Increased restrictions on IVF are projected to translate into additional hurdles for older women who choose to sequentially prioritize career over family planning as well as the continuation of high Medicaid costs for births among young women. 

Legal impact

Abortion had been legal across the United States for nearly 50 years before ‘Roe v. Wade’ was overturned by the Supreme Court in 2022; thus, there is no recent data on potential outcomes for women and their children when abortion is restricted by their home state. However, there is recent evidence suggesting that reducing the amount of time during which a woman can receive an abortion increases the number of women seeking an abortion during the early stages of pregnancy - an unintended consequence of reduced abortion access. Previously, most abortions in Texas, for example, were performed at around eight weeks of gestation, giving women more time to consider their options and life situations. Now, Texas restricts abortion to six weeks of gestation, and pregnancy can only be confirmed via ultrasound (and abortion then performed) at five weeks of gestation [47]. Having only one week between the earliest fetal ultrasound imaging and the end of the state-legal abortion limit has pushed the decision-making process toward earlier abortions. The alternative is abortion tourism to another state later in pregnancy, which can be cost-prohibitive to those who are already facing financial hardship.

Despite the cost of travel, more abortions are being performed in states that offer less restrictive abortion laws [48]. Illinois, for example, has seen an increase in the number of women traveling from other states to have an abortion - a direct consequence of neighboring Missouri tightening restrictions [48]. Tighter restrictions have generally increased demand for abortion in states that offer more complete and flexible women’s healthcare. Abortion restrictions have also increased the number of monetary donations to organizations funding travel and additional expenses for those in need of access to services out of state. In response, Missouri is attempting to restrict its residents from traveling out of state to receive abortion care [49]. This raises the question of whether states will attempt to prohibit their citizens from traveling to receive other medical procedures including experimental treatments. On July 15, 2022, the House of Representatives passed several bills that would restore abortion access nationwide and allow travel for abortion access. However, those have stalled in the Senate [9].

The proposed restrictions by many states raise the question of whether state law can supersede federal law through its institutions, including the food and drug administration (FDA). All drugs used for medication abortion are already legal at the federal level thanks to FDA approval. Most abortions in the early stages of pregnancy are medication abortions, and, during the ongoing COVID-19 pandemic, access to the appropriate drugs has been regulated via telehealth visits [50]. There is no legal precedent for making FDA-approved medications illegal at the state level. It is questionable whether the Supreme Court would allow such a maneuver, as it could exacerbate health inequities unrelated to pregnancy [51]. There have already been cases where pharmacists have refused to fill prescriptions for medications that can be used for abortion but are also needed to treat other health conditions. Methotrexate is one of those drugs that can be used to treat cancer and autoimmune diseases but also to induce abortion [5]. The HHS has recently issued guidance on the discriminatory statute of those decisions because denials have only been experienced by women of childbearing age [52]. If it turns out that individual states are given leeway to regulate the distribution of FDA-approved pharmaceuticals, patients may have to travel out of state to receive essential medications, not just for abortion but also for other healthcare needs.

Physicians are also concerned about the consequences of making decisions on abortion in instances where the mother’s life is in danger. It has been pointed out that laws providing exceptions to saving the life of the mother fail to offer clear guidance on when an abortion would be legally permissible. There are cases in which the danger increases as the pregnancy progresses, causing ambiguity as to what may be considered imminent danger before an abortion can be legally performed. Several states have made newly illegal abortions a felony. Because of that, physicians now fear that making a good faith effort to determine whether an abortion is medically necessary to save a woman’s life could result in criminal charges or loss of their medical licensure due to the element of interpretation [53].

States also have a reasonable interest in restricting abortion because of a natural interest in preserving the lives of their citizens, including the unborn. The unborn are therefore protected by federal law. If a pregnant female is attacked or murdered, the death of her fetus can be charged as a separate crime or used to enhance sentencing for her attacker [54]. The same standard could arguably be applied to women who procure abortions and those who support or provide them. Because a fetus has a unique DNA profile, it is considered a separate entity from its mother and therefore its own living entity that the state has a reasonable interest in protecting. Under the 14^th^ amendment of the US Constitution, everyone is entitled to equal protection under the law; permitting abortion to proceed seemingly counteracts that protection, some have argued [55].

Twenty-seven states currently have laws that penalize physicians for performing abortions [20]. Most of the states are predominantly rural, like Wyoming, which is already suffering a physician shortage [56]. With only 9% of the nation’s physicians practicing in rural communities, restrictive policies on abortion could exacerbate this shortage by deterring physicians even more [57]. The threat of both criminal charges and loss of medical licensure could exacerbate the shortage of healthcare workers in states that impose abortion restrictions. As new graduate physicians decide where to practice, those who believe that women should be allowed to make their own decisions about whether to continue a pregnancy are likely to choose to practice in states that align with their own values. These dynamics are likely to increase physician shortages in many rural states with a restrictive outlook on abortions. A large, well-known medical recruiting firm operating in one of the more restrictive states recently reported that 20 obstetrician-gynecologists turned down a position because of the legal nature of the abortion landscape [11].

The various definitions of fetal life are one of the drivers of a shifting abortion landscape. If life begins at fertilization, the implementation of abortion restrictions could protect the lives of children. The fetus is arguably part of a vulnerable population that needs protection from harm *in utero*. Depending on the perspective, supporting abortion, even early on, can be synonymous with the termination of human life and not just an amorphous cellular conglomerate. Increasing restrictions can also facilitate discontinuing the use of abortion as a means of birth control [58]. Following the Supreme Court’s decision to overturn ‘Roe v. Wade’, Texas law now considers life to begin at fertilization. The rights of the unborn fetus have come into question with the case of a 34-week pregnant Texas woman who is contesting a traffic ticket for driving in the carpool lane “alone”. She is contesting the recognition of the personhood of her unborn as a passenger to be allowed to drive in the carpool lane [59].

In July 2022, a case in Ohio raised awareness regarding the potential impact of an absent rape exception within more stringent abortion restrictions. An Ohio law from 2019 bans abortions at six weeks coinciding with the earliest detection of a fetal heartbeat [60]. A 10-year-old rape victim was found to be six weeks and three days pregnant and therefore unable to receive an abortion within her home state, requiring her to travel to a neighboring state with a less restrictive abortion landscape for abortion care [60].

Ambiguity on what falls under the umbrella of abortion restrictions follows suit on Plan B (aka morning after pill). Even though this medication is intended to prevent pregnancy in the first place, it can be interpreted differently in terms of its clinical use as a family planning tool. A hospital in Kansas City, Missouri temporarily stopped providing Plan B for patients due to the unclear legal implications for providers [61].

Financial impact

Restrictions or limitations on abortion access will inevitably result in the continuation of unwanted pregnancies with a range of long-term effects on the birthing individual. The Turnaway Study found that women forced to continue an unwanted pregnancy are more likely to remain in unhealthy relationships, suffer mental and physical health consequences, live in poverty, and have lower life satisfaction [8]. Being pressured to maintain an unwanted pregnancy can have profound effects on a person’s mental health due to the drastic life changes that come with motherhood such as an impact on her career and finances, and the sudden responsibility of caring for a new life. A study comparing poverty inequalities between single mothers and fathers found that 24.3% of single mothers were in a state of poverty crisis (at or below 100% of the federal poverty level), whereas only 6.9% of single fathers fit that category [62]. Mothers are also at a disadvantage when it comes to advancement in their careers. Childless women are 8.2 times more likely to be recommended for promotion than mothers [37].

Additionally, strict abortion regulations will likely require states and local governments to reallocate funding for the expansion of neonatal healthcare services and programs [63]. Currently, many states are imposing near-total abortion bans, outlawing abortion based on gestation stage. A reasonable outcome of stricter gestation laws is increased neonatal morbidity and a rise in congenital conditions. Prenatal tests, such as ultrasound and amniocentesis indicating genetic anomalies and anatomical deficits, do not take place until after the gestational limits imposed by many states [32,63]. This means that women living in these states will be forced to continue their pregnancy even if the child will suffer from serious congenital disorders and anomalies requiring lifelong treatment and care [64]. As a result, a significant increase in infants born with congenital conditions that may require costly neonatal intensive and even lifelong care must be expected by state budgets. For women in abortion-restrictive states, this will likely translate into a disproportionate dependency on the state’s Medicaid system as well as federal disability funding.

Religious impact

Abortion has long been a subject of both religious as well as political debate. The Republican Party listed banning abortion and protecting fetal life as part of its 2016 national platform (the most recent available). While the Democratic party did not include the issue of abortion most recently, there is a historical connection to a pro-choice platform [65]. Republicans argue that abortion destroys a unique life and that adoption is a better option in cases of unwanted pregnancy. Democrats argue that women know more about their own situations and whether carrying a pregnancy to term fits with their goals, values, and circumstances. The world’s major religions, and even subgroups within them, are split on the issue. Strictly pro-life religions and denominations include the Roman Catholic Church, the Southern Baptist Convention, and Hinduism. Judaism, Presbyterianism, and the United Church of Christ advocate for abortion access with few restrictions [66].

A Jewish community in Florida is currently suing the state on grounds that the ban on allowing abortion after 15 weeks is a breach of religious freedoms. According to Jewish law, abortion is necessary if the pregnancy negatively affects the woman’s health as defined by either mental or physical well-being. By not permitting abortions in this or similar cases, religious beliefs may be curtailed, which may conflict with the First Amendment to the US Constitution [67]. While the intent of giving the power over to the states may be to settle the long-standing debate over abortion, this may violate religious freedom and pit one religion’s beliefs against another’s. With the state endorsing the beliefs of some but not all religions, the separation of church and state becomes an issue in the debate surrounding abortion.

Recommendations

To stress the importance of unbiased information in decision-making, we advocate for the initiation of a comprehensive study on maternal health and socioeconomic outcomes in response to the recent decision by the Supreme Court to shift the responsibility of abortion legislation back to the individual states. Such as study must be stratified by SDOH as defined and further developed by the Centers for Disease Control and Prevention (CDC) [68]. This process will ensure a differentiated analysis taking into account the vast differences in living circumstances across the country. In addition to women's health, the study must also consider infant mortality, child-developmental milestones, as well as the costs of entitlement and insurance programs.

Furthermore, the study should examine the potential effects on access to reproductive palliative care, maternal care, labor and delivery services, and postpartum care due to a shortage of OB/GYN specialists or healthcare providers. One component of the study should assess both short- and long-term risks associated with unsafe abortion and pregnancy, such as morbidity and mortality. There is a lack of data on the impact of legislative interference on doctor-patient relationships due to the potential legal consequences of physicians as mandated reporters [69]. Interviewing newly graduated and relocating physicians to find out if abortion policies are a consideration when deciding where to practice could be one strategy to collect data. In addition, the study should look at migration patterns and whether states face changes in population numbers and demographics.

There is also a lack of data on the influence that state-based abortion policies can have on the ongoing regional healthcare capacity challenges. It is plausible that hospitals will need to accommodate a larger influx of birthing individuals without being properly staffed, further exacerbating ongoing limitations such as hospital bed capacity, staffing, and general medical resources. The proposed study should therefore include statistics on prenatal care and the incidence of babies born not receiving prenatal care or screening. The research should also include information about how budgets for education and the foster care system are impacted by abortion policy. Analyses of all of these impacts could provide critical information for future planning and strategies for the expansion of healthcare infrastructure in light of the current regulatory shift.

## Conclusions

The recent decision of the US Supreme Court to return abortion legislation to individual states will profoundly impact health equity across the country. Even in the few months since ‘Roe v. Wade’ has been overturned, cases have emerged highlighting the broad consequences of this decision. From rural to urban America, the cascading effects are seen and felt by physicians, patients, state governments, and corporations. As rural states, which are more likely to restrict abortion, continue to face physician shortages, they must consider how their policies will exacerbate those shortages and increase the inequality of care for their residents.

The US Congress, in accordance with its unifying role, will need to consider whether unequal health outcomes are worthy of concern and whether to establish standardized abortion laws. The study described and recommended herein could serve as the basis for an informed decision-making process going forward.
